# The Citrus transcription factor, CitERF13, regulates citric acid accumulation via a protein-protein interaction with the vacuolar proton pump, CitVHA-c4

**DOI:** 10.1038/srep20151

**Published:** 2016-02-03

**Authors:** Shao-jia Li, Xue-ren Yin, Xiu-lan Xie, Andrew C. Allan, Hang Ge, Shu-ling Shen, Kun-song Chen

**Affiliations:** 1 College of Agriculture & Biotechnology, Zhejiang University, Zijingang Campus, Hangzhou 310058, P.R. China; 2 Zhejiang Provincial Key Laboratory of Horticultural Plant Integrative Biology, Zhejiang University, Zijingang Campus, Hangzhou 310058, P.R. China; 3 The State Agriculture Ministry Laboratory of Horticultural Plant Growth, Development and Quality Improvement, Zhejiang University, Zijingang Campus, Hangzhou 310058, P.R. China; 4 New Zealand Institute for Plant & Food Research Limited, Private Bag 92169, Auckland, New Zealand; 5 School of Biological Sciences, University of Auckland, Auckland, New Zealand

## Abstract

Organic acids are essential to fruit flavor. The vacuolar H^+^ transporting adenosine triphosphatase (V-ATPase) plays an important role in organic acid transport and accumulation. However, less is known of V-ATPase interacting proteins and their relationship with organic acid accumulation. The relationship between V-ATPase and citric acid was investigated, using the citrus tangerine varieties ‘Ordinary Ponkan (OPK)’ and an early maturing mutant ‘Zaoshu Ponkan (ZPK)’. Five V-ATPase genes (*CitVHA*) were predicted as important to citric acid accumulation. Among the genes, *CitVHA-c4* was observed, using a yeast two-hybrid screen, to interact at the protein level with an ethylene response factor, CitERF13. This was verified using bimolecular fluorescence complementation assays. A similar interaction was also observed between *Arabidopsis* AtERF017 (a CitERF13 homolog) and AtVHA-c4 (a CitVHA-c4 homolog). A synergistic effect on citric acid levels was observed between V-ATPase proteins and interacting ERFs when analyzed using transient over-expression in tobacco and *Arabidopsis* mutants. Furthermore, the transcript abundance of *CitERF13* was concomitant with *CitVHA-c4. CitERF13* or *AtERF017* over-expression leads to significant citric acid accumulation. This accumulation was abolished in an *AtVHA-c4* mutant background. ERF-VHA interactions appear to be involved in citric acid accumulation, which was observed in both citrus and *Arabidopsis*.

Organic acids play a pivotal role in plant growth and development; in *Arabidopsis*, organic acid was observed to be involved in germinating seedlings^1^. In addition, they participate in a multiplicity of pathways in plant metabolism and are related to metal-resistance^2^. In fruit, organic acids contribute to flavor, one of the most important fruit quality traits, thus affecting consumer preference. Organic acids also play a crucial role in fruit nutrition and fruit beverage quality^3^. Concentrations of organic acids are affected by genetic, developmental and environmental stimuli. Among different crops, acidity varies within species or cultivars^4^. During fruit development, organic acids usually accumulate at early stages and then decrease towards fruit maturity^5^. Environmental stimuli, such as light and temperature, also influence fruit organic acid metabolism^6^.

Organic acids, including citric, malic, tartaric, succinic and oxalic acids, are present in various fruits, providing characteristic flavors. In citrus fruits, the predominant organic acid is citric acid. The accumulation of organic acids in fruit is determined by the balance of biosynthesis, degradation and vacuolar storage^7^. Most research has focused on fruit citric acid biosynthesis and degradation. For citric acid biosynthesis, the activity of mitochondrial citrate synthase (mtCS) was observed to be positively correlated with citrate accumulation in citrus^8^. However, some reports suggest that the activity and expression of mtCS is not responsible for differences in citrate content between low- and high-acid cultivars^9^,^10^. The degradation pathway of citric acid has also been investigated, suggesting that cytosolic aconitase (ACO) and NAD-isocitrate dehydrogenase (NAD-IDH) activities control citric acid degradation during lemon fruit (*Citrus limettioides* Tan., low acid; *Citrus limon* var. Eureka, high acid) development^11^,^12^. In addition, *ACO* and *IDH, CsGAD1/2* and *CitGAD4* are involved in citric acid utilization in orange and mandarin fruit (*Citrus sinensis* cv. Anliu; C. sinensis cv. Niuher; *C. unshiu* cv. Guoqing No.1^13^) and hot air driven citric acid degradation in ‘Ponkan’ fruit (*C. reticulata* Blanco cv. Ponkan^14^). Following citric acid biosynthesis and degradation, vacuolar storage is important as the flavor of citrus fruit is determined by the concentration of citric acid in vacuole.

Vacuolar accumulation of citric acid is thought to be controlled mainly by vacuolar H^+^-ATPases^15^. The vacuolar H^+^ transporting adenosine triphosphatase (V-ATPase), also known as the ‘proton pump’, is a multi-subunit enzyme using the energy released from the hydrolysis of ATP to pump protons into the vacuolar lumen, thereby creating an electrochemical H^+^ gradient which is the driving force for a variety of transport events including ions and metabolites, such as organic acids^16^. The expression of V-ATPase subunit A is associated with loquat fruit acidity, exhibiting higher expression in low-acid loquat fruit ‘Changhong 3’^17^. Similar results were observed in some other fruit, such as grapes, peaches and apples^18^,^19^,^20^. In citrus, investigation of the vacuoles of the acid lime juice cell suggests citrate uptake can be accounted for by direct primary active transport mechanisms involving ATP^21^. In addition, the studies in acidic lemon and limes indicated that citric acid accumulation was accompanied by a large influx of protons mediated by the vacuolar H^+^-ATPase. This influx of protons reduces the vacuolar pH and provides a driving force for additional citric acid uptake^15^,^22^. Thus, there is evidence that V-ATPase contribute to citric acid content in citrus fruit. However, the mechanisms were relatively unknown, and regulation of the V-ATPase protein complex is not well understood.

In this current study, a novel ethylene response factor (*ERF*), CitERF13, was shown by yeast two-hybrid assay to interact with the citrus vacuolar proton pump (CitVHA-c4) at the protein-protein level. The correlation of gene expression of *CitERF13* and *CitVHA-c4* (as well as other coding genes for V-ATPase), with citric acid content was analyzed in two cultivars of Ponkan, a sweet variety of tangerine. The functional characterization of *CitERF13* and *CitVHA-c4* was analyzed by transient overexpression in tobacco (*N. tabacum)* and mutants of *Arabidopsis*. The results suggest that CitERF13 is involved in citric acid accumulation, via protein-protein interaction with CitVHA-c4.

## Results

### Changes in Organic Acid content during Fruit Development

**Figure 1:**
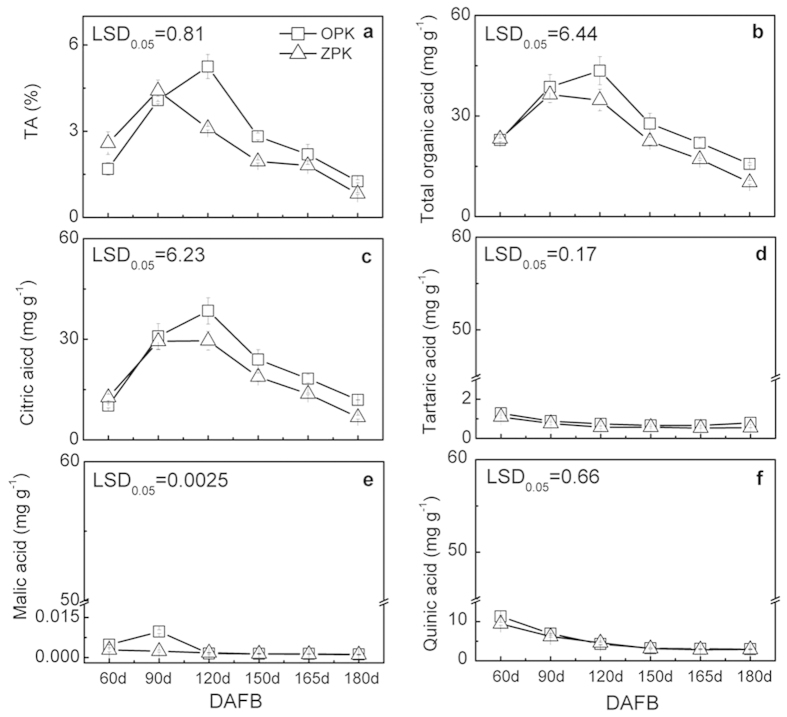
Changes in the contents of titratable acid (TA, (**a**)), total organic acids (**b**), citric acid (**c**), tartaric acid (**d**), malic acid (**e**) and quinic acid (**f**) in flesh of Pokan fruits during fruit development, DAFB means day after full blossom. Error bars on each column indicate ±SE from three biological replicates. LSDs represent least significant differences at 0.05.

Organic acids, including citric, tartaric, malic and quinic acid, were measured during Ponkan fruit development (Fig. 1). The results indicate that citric acid is the major component of ‘Ponkan’ fruit organic acids, with 11.90 mg g^−1^ at maturity in OPK fruit (180 DAFB) (Fig. 1c). Tartaric acid, malic acid and quinic acid contents were lower at the same stage in OPK fruit, with 0.79 mg g^−1^, 1.12 mg g^−1^ and 2.97 mg g^−1^, respectively (Fig. 1d–f).

As citric acid is the major organic acid in ‘Ponkan’ fruits, changes in citric acid content were highly correlated with TA, during fruit development. In both of ZPK and OPK fruit, TA value, total organic acids and citric acid content increased during fruit early developmental stages (peaked at 120 DAFB in OPK fruit) and decreased afterwards till commercial maturity stage (180 DAFB) (Fig. 1a–c). In contrast, the tartaric acid, malic acid and quinic acid contents remained from 60 DAFB to 180 DAFB, with slight changes during development (Fig. 1d–f).

When comparing the two cultivars, the dynamics of organic acid and TA changes were similar between OPK and ZPK. The main difference between OPK and ZPK, was the value of TA and content of total organic acid and citric acid. As shown in Fig. 1, OPK had higher TA, total organic acid and citric acid which peaked with 5.25%, 43.50 mg g^−1^ and 38.47 mg g^−1^ at 120 DAFB; while ZPK peaked earlier at 90 DAFB with value of 4.41%, 36.37 mg g^−1^ and 29.39 mg g^−1^, respectively. After peak period, TA, total organic acid and citric acid decreased in both cultivar s, and the differences between two cultivars were constant till maturity, with value of −1.25%, 15.66 mg g^−1^ and 11.9 mg g^−1^ in OPK and was lower in ZPK of −0.82%, 10.20 mg g^−1^ and 6.74 mg g^−1^ (Fig. 1a–c)

### Expression of V-ATPase Genes during Fruit Development

**Figure 2:**
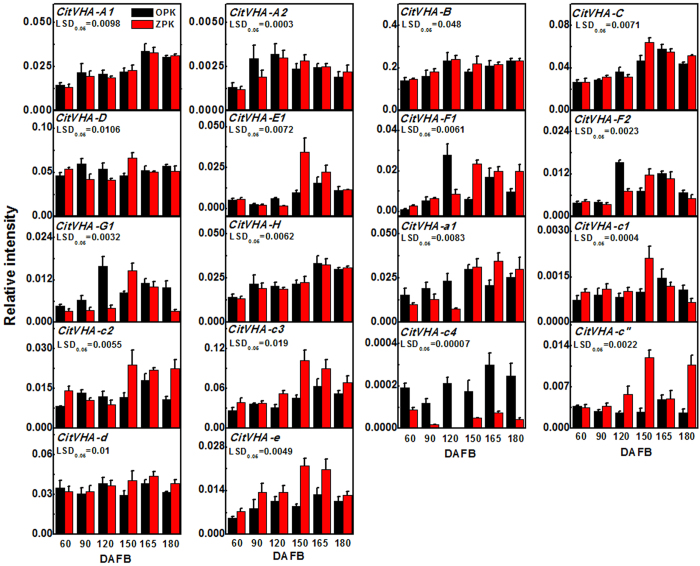
Expression of the V-ATPase genes in flesh of Ponkan fruits during fruit development, DAFB means day after full blossom. Error bars on each column indicate ±SE from three biological replicates. LSDs represent least significant differences at 0.05.

V-ATPases have been associated with organic acid accumulation. However, the role of V-ATPases in citric acid content in citrus fruit is limited. According to the previous nomenclature (Sze *et al*., 2002^23^), 18 V-ATPase genes were identified by blast using *Arabidopsis* V-ATPase genes in the citrus genome database (http://www.citrusgenomedb.org). The 18 V-ATPase genes were designed as *CitVHA-A1* (clementine0.9_005081m), *CitVHA-A2* (clementine0.9_007969m), *CitVHA-B* (clementine0.9_008666m), *CitVHA-C* (clementine0.9_013205m), *CitVHA-D* (clementine0.9_019158m), *CitVHA-E1* (clementine0.9_020736m), *CitVHA-F1* (clementine0.9_024363m), *CitVHA-F2* (clementine0.9_025353m), *CitVHA-G1* (clementine0.9_026050m), *CitVHA-H* (clementine0.9_030560m), *CitVHA-a1* (clementine0.9_002488m), *CitVHA-c1* (clementine0.9_023951m), *CitVHA-c2* (clementine0.9_023956m), *CitVHA-c3* (clementine0.9_023909m), *CitVHA-c4* (clementine0.9_023943m, previously known as *CitVATP-c1*, AB024274.1), *CitVHA-c”* (clementine0.9_023193m), *CitVHA-d* (clementine0.9_014414m), *CitVHA-e* (clementine0.9_027237m). Gene expression analysis indicated that *CitVHA* genes differentially expressed during fruit development (Fig. 2).

Using OPK as a reference, eight *CitVHA* genes exhibited increasing expression pattern during fruit development; *CitVHA-A1, CitVHA-A2, CitVHA-C, CitVHA-E1, CitVHA-H, CitVHA-a1, CitVHA-c3* and *CitVHA-e*. Four genes, *CitVHA-A2, CitVHA-F1, CitVHA-F2* and *CitVHA-G1*, showed a peak in expression at 120 DAFB; while some members showed more constitutive expression during development, such as *CitVHA-D* and *CitVHA-d* (Fig. 2).

In parallel with citric acid content, differential expression of some *CitVHA* genes were also observed from two cultivars, such as *CitVHA-F1, CitVHA-F2, CitVHA-G1* and *CitVHA-a1* were relatively higher expressed at early development stages (60–120 DAFB) in OPK fruit than in ZPK fruit (Fig. 2). In contrast, several *CitVHA* genes, such as *CitVHA-E1, CitVHA-c3, CitVHA-c”* and *CitVHA-e*, were highly expressed in ZPK fruit at late development stage (Fig. 2). Of interest was *CitVHA-c4*, which showed substantially higher expression in OPK fruit than in ZPK fruit (Fig. 2). Thus, *CitVHA-c4*, could be a candidate responsible for differences in citric acid content in ‘Ponkan’ fruit.

### A transcription factor, CitERF13, interacts with CitVHA-c4

**Figure 3:**
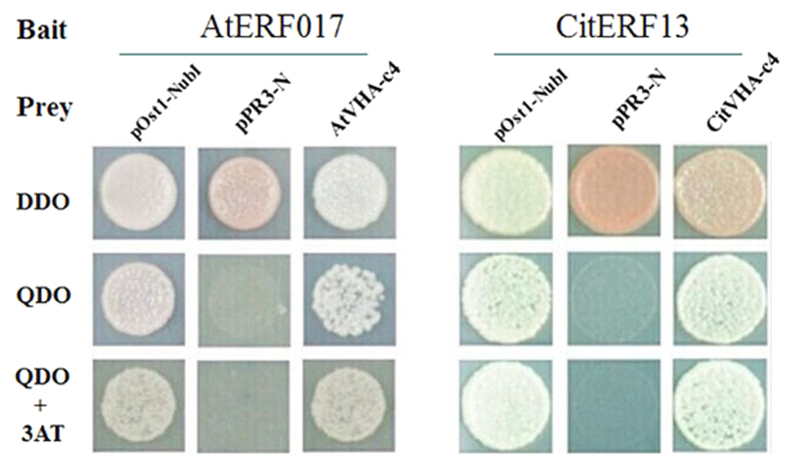
Yeast two-hybrid assays show that AtERF017 interacts with AtVHA-c4 and CitERF13 interacts with CitVHA-c4. AtERF017 and AtVHA-c4 are homologs of CitERF13 and CitVHA-c4 in *Arabidopsis*. Liquid cultures of double transformants are plated at OD_600_ = 0.1 dilutions of the cultures on synthetic dropout selective medium: (1) SD medium lacks Trp and Leu (DDO); (2) SD medium lacks Trp, Leu, His and Ade (QDO); (3) SD medium lacks Trp, Leu, His, Ade, and was supplemented with 10 mM 3-amino-1,2,4-triazole (QDO+3AT). Protein-protein interactions were determined on QDO and QDO + 3AT. pOst1-NubI, positive control; pPR3-N, negative control.

A transcriptome-wide screening experiment was performed to identify potential proteins that could interact with the closest *Arabidopsis* homologue to CitERF13, AtERF017 (At1g19210), using the commercial yeast two-hybrid library from DUALhunter systems Biotech (Switzerland). A protein was identified which shown to be AtVHA-c4 (At1g75630), that interacted with AtERF017 (Fig. 3). Phylogenetic analysis indicated that AtVHA-c4 is a homolog of CitVHA-c4 (Fig. S2). Thus, it is proposed that *ERF* transcription factor may act novel regulator of citric acid metabolism via binding to the V-ATPase.

Within the *CitAP2/ERF* family, an ethylene response factor (*CitERF13*, isolated by Xie *et al*.^24^ was found as the closest homolog of AtERF017, based on phylogenetic analysis (Fig. S2). Thus, potential protein-protein interaction between CitERF13 and CitVHA-c4 was analyzed. Similar to AtERF017, CitERF13 could also interact with CitVHA-c4 (Fig. 3). Moreover, further analyses indicated that such interaction only occurred between CitERF13 and CitVHA-c4, but not the others c units, including CitVHA-c1, CitVHAc2, CitVHA-c3 and CitVHA-c” (Fig. S3). This result, suggests that CitERF13 and AtERF017 participate in citric acid regulation via a specific interaction with one subunit of the V-ATPase.

**Figure 4:**
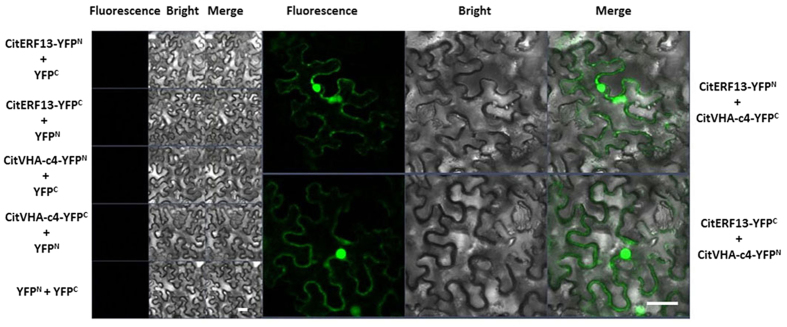
*In vivo* interaction between CitERF13 and CitVHA-c4, using BiFC. BiFC analysis for interaction between CitERF13 and CitVHA-c4. N- and C-terminal fragments of YFP (YFP^N^ and YFP^C^) were fused to the C terminus of CitERF13 and CitVHA-c4, respectively. Combination of YFP^C^ or YFP^N^ with corresponding CitERF13 or CitVHA-c4 constructs were used as negative controls. Fluorescence of YFP represents protein-protein interaction. The bars indicated the length of 50 μm.

To further confirm the interaction between CitERF13 and CitVHA-c4 and to verify the *in vivo* interactions in planta, bimolecular fluorescence complementation assays were performed. The results showed that the negative combination, such as CitERF13-YFP^N^/YFP^C^, YFP^N^/CitERF13-YFP^C^, CitVHA-c4-YFP^N^/YFP^C^, and YFP^N^/CitVHA-c4-YFP^C^ and YFP^N^/YFP^C^ did not produce any detectable fluorescence signal, while co-expression of CitERF13-YFP^N^ and CitVHA-c4-YFP^C^ or CitVHA-c4-YFP^N^ and CitERF13-YFP^C^ gave strong signals both in the nucleus and at the tonoplast, such fluorescence detection indicated protein-protein interaction between CitERF13 and CitVHA-c4 and further supported yeast two hybrid results (Fig. 4).

### Association of *CitERF13* expression and citric acid during fruit development

**Figure 5:**
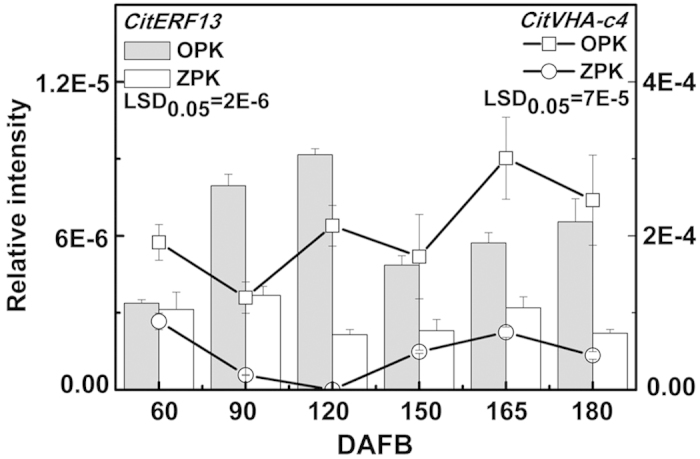
Expression of the *CitERF13* and *CitVHA-c4* genes in flesh of Pokan fruits during fruit development, DAFB means day after full blossom. Error bars on each column indicate ±SE from three biological replicates. LSDs represent least significant differences at 0.05.

In order to confirm the relationship between *CitERF13* and citric acid, the expression of *CitERF13* was studied during fruit development in OPK and ZPK. The results indicated that *CitERF13* had a similar expression pattern to *CitVHA-c4*, which was more abundant in the high-acidity OPK fruit during fruit development (Fig. 5). Furthermore, other genetically unrelated citrus material was checked. It was found that *CitERF13* exhibited higher expression in the high-acidity ‘Gaocheng’ Mandarin than in low-acidity ‘Satsuma Mandarin’^25^ (Fig. S4).

### Effects of *CitERF13* and *CitVHA-c4* on in citric acid accumulation

**Figure 6:**
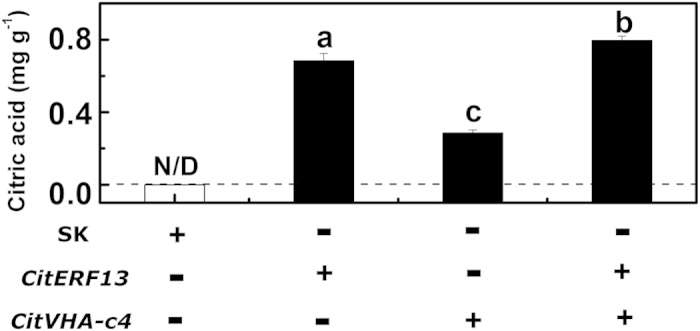
Transient overexpression of *CitERF13* and *CitVHA-c4* in *N. tabacum* leaves. Both *CitERF13* and *CitVHA-c4* genes were driven by the CaMV 35S promoter. SK represents empty vector. Citric acid was analyzed at 5 d after infiltration, with three biological replicates. Different letters indicate a significant difference (*p* < 0.05), which were calculated using Student’s *t*-test. +, present of the construct; −, absence of the construct.

As citrus is a perennial fruit, thus transient overexpression analyses were conducted *N. tabacum* leaves to verify *CitERF13* and *CitVHA-c4* function^26^. Compared with empty vector control, transient overexpression of *CitERF13* and *CitVHA-c4* significantly increased the citric acid accumulation in tobacco leaves. Citric acid in *N. tabacum*, transformed with *Agrobacterium* harboring the empty vector alone, was not detectable. With transformation of the leaves with either *CitERF13* or *CitVHA-c4* alone, the citric acid in *N. tabacum* leaves reached 0.52 mg g^−1^ and 0.25 mg g^−1^, respectively (Fig. 6). Moreover, a combination of *CitERF13* and *CitVHA-c4* accumulated more citric acid, reaching 0.68 mg g^−1^ (Fig. 6).

**Figure 7:**
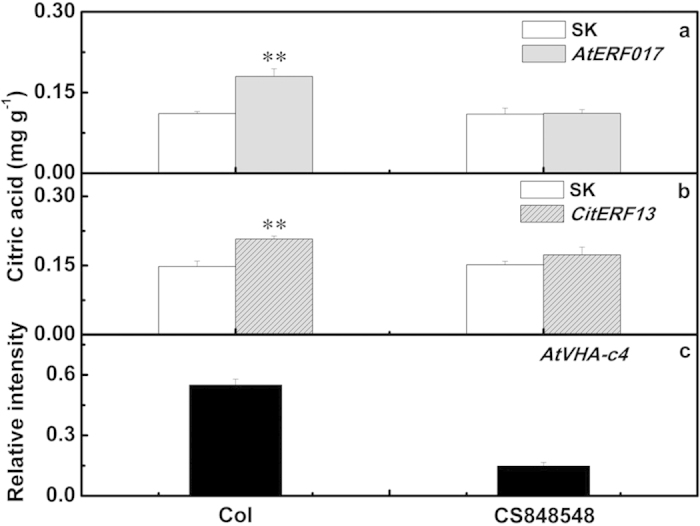
Transient overexpression of *AtERF017* and *CitERF13* in *Arabidopsis* leaves. CS848548 is a mutant for *AtVHA-c4* and was obtained from TAIR. (**a**,**b**) Citric acid was analyzed at 3 d after infiltration, with three biological replicates. The statistical significance of differences was calculated using Student’s *t*-test. Asterisks *indicate significant differences (*p* < 0.05), double asterisks **indicate significant differences (*p* < 0.01). (**c**) AtVHA-c4 mRNA abundance in wild type and CS848548. *AtERF017* and *CitERF13* gene was driven by the CaMV 35S promoter. SK represents empty vector.

The function of the *ERF*, and its interaction with the V-ATPase, were further confirmed in *Arabidopsis AtVHA-c4* mutant. An *AtVHA-c4* mutant (CS548548) was obtained from TAIR, and real-time PCR analysis indicated the endogenous AtVHA-c4 had relatively low expression in the mutant (CS848548), with approximately 75% reduction of that in WT plants (Fig. 7c). Transient overexpression of *AtERF017* significantly increased citric acid accumulation; in wild type Col *Arabidopsis* leaves, with transient expression of *AtERF017*, citric acid reaches 0.18 mg g^−1^, compared with only 0.11 mg g^−1^ in the leaves infiltrated with empty vector. However, in the *AtVHA-c4* mutant CS848548, *AtERF017* transient overexpression failed to enhance citric acid accumulation, compared with empty vector (Fig. 7a). Similar results were found using *CitERF13*, the homolog of *AtERF017*, which also exhibited significant enhancement of citric acid level in *Arabidopsis* leaves (Fig. 7b).

## Discussion

Most organic acids are found in the plant vacuole, which is considered as the main reservoir of these metabolites^27^. As one of the most important enzymes, the V-ATPase, therefore plays a very important role in organic acid accumulation. The V-ATPase is a large multimeric enzyme organized in two domains, V_1_ and V_0_. The peripheral V_1_ domain is responsible for hydrolysis of ATP and was comprised of eight subunits (A-H), while V_0_ domain is composed of six different subunits a, d, c, c’ (absent in plants), c”, e, which function on translocation of protons across the membrane^28^,^29^. Each subunit has dominant functions for some specific processes, eg. *VHA-E1* plays an essential role in maintaining a functional secretory system during somatic development^30^; *CsVHA-c1* and *CsVHA-c2* were essential elements of mechanisms involved in adaptation of cucumber plants to copper toxicity^31^; *MdVHA-A* and *MdVHA-B* from apple were observed to be involved in drought tolerance^32^,^33^. However, most research has focused on transcriptional response of VHA genes in response to environmental stimuli and the functional characterization of each subunit. The linkage between V-ATPases and transcription factors has not been investigated.

Here, 18 genes encoding subunits of V-ATPase were isolated from the citrus genome database. In order to relate gene expression with organic acid levels, OPK and ZPK were chosen as material, as ZPK was considered as a mutant bud sport of OPK, thus ZPK and OPK should had similar genetic backgound. It is interesting that ZPK and OPK had a very similar profile of organic acid change, but ZPK had lower organic acid (mainly citric acid) than OPK fruit. Gene expression suggested indicated that *CitVHA-F1, CitVHA-F2, CitVHA-G1, CitVHA-a1* and *CitVHA-c4* were associated with the citric acid content in OPK and ZPK. Similar results were reported in ‘sweet’ and ‘sour’ lemon, in which expression of the H^+^-ATPase AHA10 is higher in sour lemon, and may be involved in citric acid biosynthesis and accumulation in juice sac cells^34^. These results provided further molecular evidence for the role of V-ATPase in citric acid, and those five genes (*CitVHA-F1, CitVHA-F2, CitVHA-G1, CitVHA-a1* and *CitVHA-c4* might be the key dominant unit for different acidity in citrus fruit.

*CitVHA-c4* was constitutively higher in expression in OPK fruit, which correlates with higher citric acid content. A similar correlation of *CitVHA-c4* expression was also observed using high-acid ‘Gaocheng’ (GC) and the low-acid ‘Satsuma Mandarin’ (SM) (Fig. S4). Transient over-expression of *CitVHA-c4* in *N. tabacum* leaves significantly elevated citric acid accumulation. Thus, it is proposed that *CitVHA-c4* is an important unit for citric acid accumulation, which contributes to citrus fruit acidity and flavor.

One of most interesting results from this study is the novel protein-protein interaction observed between CitERF13 and CitVHA-c4. Such an interaction was further confirmed in *Arabidopsis* between AtERF017 (a homolog of CitERF13) and AtVHA-c4 (a homolog of CitVHA-c4), suggesting that the interaction between AP2/ERF and V-ATPase might be conserved within plants. AP2/ERFs are a large plant specific transcription factor family, which are involved in many aspects of plant development and stress response^35^,^36^. In fruit, various AP2/ERF genes were characterized to be involved in fruit ripening^37^,^38^ and quality aspects (eg. Carotenoid^39^; texture^40^; taste^41^,^42^). No association of an ERF and V-ATPase has been reported. However, some other well-known proteins function via the V-ATPase, such as the protein kinase SOS2 which promotes salt tolerance by interacting with VHA-B subunits^43^. The regulatory 14-3-3 proteins are activators which interact with the A-subunit of the V-ATPase after blue light treatment^44^. In addition, different subunits could also interact with each other, such as subunit c and a^45^. Therefore, citrus CitERF13 and *Arabidopsis* AtERF017 appear to have a novel interaction with the V-ATPase.

Using GFP tags *CitERF13* and *CitVHA-c4* were predicted to have different subcellular localizations. It has been reported in *Arabidopsis* that the ethylene-responsive element binding protein (AtEBP) may move from the nucleus to the cytosol via protein-protein interaction with ACBP4^46^; a rice SPX family protein, OsSPX4, can also reduce the targeting of OsPHR2 to the nucleus through its interaction with PHR2 when Pi is sufficient^47^. In our citrus study, a protein-protein interaction between CitERF13 and CitVHA-c4 was verified using BiFC assays. This indicated a strong signal in both the tonoplast and nucleus. Thus, interaction between CitERF13 and CitVHA-c4 may shift their sub-cellular localization. Importantly, *CitERF13* and its homolog *AtERF017*, both exhibited the ability to enhance citric acid accumulation. Transient over-expression of *CitERF13* in tobacco triggered citric acid accumulation. Moreover, combinatorial effects of *CitERF13* and *CitVHA-c4*, as well as *AtERF017* and *AtVHA-c4*, were observed. A combination of *CitERF13* and *CitVHA-c4* infiltrated into tobacco resulted in an increment (with significance), compared with *CitERF13* and *CitVHA-c4* infiltrated individually. These results suggested that *CitERF13* and *CitVHA-c4* work as partners in citric acid accumulation. Both of *AtERF017* and *CitERF13* was failed to trigger citric acid accumulation in leaves of CS838548, where *AtVHA-c4* was significantly repressed. Taken together, these results suggest *CitERF13*, as well as *Arabidopsis AtERF017*, are novel regulators on citric acid accumulation, via the V-ATPase c unit.

In conclusion, our results indicated that in citrus, *CitVHA-F1, CitVHA-F2, CitVHA-G1, CitVHA-a1* and *CitVHA-c4* are related to the citric acid accumulation. A novel protein-protein interaction was observed between CitERF13 and CitVHA-c4, with such a complex linking *CitERF13* and citric acid accumulation. Although such protein-protein interactions are rarely reported in plants, the present results showed similar interactions between AtERF017 and AtVHA-c4. Thus this protein-protein interaction might be conserved for various plants.

## Methods

### Plant materials

Fruit of two cultivars of Ponkan (*C. reticulata* Blanco cv. Ponkan), named as ‘Ordinary Ponkan (OPK)’ and ‘Zaoshu Ponkan (ZPK)’, were harvested from a commercial orchard in Quzhou, Zhejiang, China. ZPK is a bud sport mutant of OPK, which in this region is early maturing. Fruits with uniform size and appearance were collected for each cultivar, at each sampling point, from six different trees. Six time points were collected at 60, 90, 120, 150, 165, and 180 days after full bloom (DAFB). The flesh was frozen in liquid nitrogen and stored at −80 °C for further experiments.

### Titratable acidity and organic acid measurement

Fruits were divided into three groups, each of four fruits, and 5 ml of juice from each group was diluted in 20 ml distilled water and then was titrated with 0.1 M NaOH to the end point at pH 8.2, according to the previous report^48^. Titratable Acidity (TA) was calculated as percentage of citric acid.

Organic acids were extracted according to the method described by Chen *et al*.^14^. Two grams of frozen flesh sample was ground to a powder in liquid nitrogen, and homogenized in 5.0 ml of ethanol (80%) at 35 °C for 20 min. The homogenate was centrifuged at 10,000 × g for 10 min, at 20 °C. The residue was extracted twice, and the supernatant was collected and supplemented with 80% ethanol to 25 ml. One milliliter extracted solution was dried under vacuum condition (Eppendorf Concentrate Plus, Germany) at 45 °C, and the residue was dissolved in 0.5 ml distilled water and filtered with Ф0.22 μm, ø13 mm water syringe filter (Shanghai Xingya Purification Material Factory, China). The filtered solution was used for further organic acids analysis.

Organic acids were analyzed by high-performance liquid chromatography (HPLC) (Waters Alliance 2695 system, Waters Corporation, USA). A 20 μl sample of eluate was injected into an ODS C18 (4.6 × 250 mm) column (Beckman, USA). The flow rate was 0.5 ml min^−1^ using 50 mM (NH_4_)_2_HPO_4_ (pH 2.7, which was adjusted by H_3_PO_4_) as the solvent. Organic acids were detected at 210 nm. The eluted peaks were detected with a Waters 2996 diode array detector (Waters Corporation, USA), and quantity of individual organic acids was calculated using peak areas of standards. All measurements for organic acids were performed with three replicates.

### RNA extraction and cDNA synthesis

Total RNA was extracted from frozen tissues according to the protocol described by Chang^49^. The genomic DNA in total RNA was degraded with RNase-free DNase I (Fermentas). A 1.0 μg DNA-free RNA was initiated for first-strand cDNA synthesis with a RevertAid™ Premium Reverse Transcriptase (Fermentas, Thermo Scientific, USA) following to the manufacturer’s protocol. Diluted cDNA was used as the template for real-time PCR analysis. RNA extraction and cDNA synthesis were performed with three biological replicates for each sampling point.

### Real-time PCR

The PCR mixture (10 μL total volume) comprised 2 μL of Lightcycler Faststart DNA Master^plus^ SYBR Green I Mix (Roche), 0.5 μL of each primer (10 mM), 1 μL of diluted cDNA and 6 μL PCR grade H_2_O. PCR was performed on a LightCycler 1.5 instrument (Roche), initiated by 5 min at 95 °C, then followed by 45 cycles of 95 °C for 10 s, 60 °C for 5 s, and 72 °C for 10 s, and completed with a melting curve analysis program. No-template controls and melting curve analyses were included in every reaction. Citrus *actin* (XM_006464503) was used as the housekeeping gene to quantify cDNA abundance^14^. The sequences of primers for *CitVHA, AtERF017* and *AtVHA-c4* were described in Table S1.

### Yeast two-hybrid assay

Protein-protein interactions were investigated in yeast with the DUAL hunter system (Dualsystems Biotech, Switzerland). Full-length coding sequences of *AtERF017* and *CitERF13* were cloned into pDHB1 vector, as bait; while full-length of AtVHA-c4 and CitVHA-c subunits were constructed into pPR3N vector, as prey. The primers used for vectors construction were described in Table S2.

All constructs were transformed in the yeast strain NMY51 according to the manufacturer’s instructions. The assays were performed with different mediums: (1) SD medium lacks Trp and Leu (DDO); (2) SD medium lacks Trp, Leu, His and Ade (QDO); (3) SD medium lacks Trp, Leu, His, Ade, and was supplemented with 10 mM 3-amino-1,2,4-triazole (QDO + 3AT). Auto-activations were tested with empty vector of pPR3-N and target genes with pDHB1, which were co-transformed in NMY51 and plated on QDO. Auto-activations were indicated by presence of colonies. Protein-protein interaction assays were performed with co-transformation of AtVHA-c4 in pPR3N with AtERF017 in pDHB1 and CitVHA-c subnuits in pPR3N with CitERF13 in pDHB1, respectively. The presence of colonies in QDO and QDO + 3AT, indicated a protein-protein interaction.

### Bimolecular fluorescence complementation assay

Full-length *CitERF13* and full-length *CitVHA-c4* were cloned into either C-terminal or N-terminal fragments of YFP vectors^50^. Primers used are listed in Supplemental Table 2. All constructs were transiently expressed in tobacco leaves by *Agrobacterium*-mediated infiltration (GV3101) according to previous reports^51^. The YFP fluorescence of tobacco leaves were imaged 3 d after infiltration using a Zeiss LSM710NLO confocal laser scanning microscope. The excitation wavelength for YFP fluorescence was 514 nm, and fluorescence was detected at 519 to 567 nm.

### Transient over-expression in tobacco and *Arabidopsis*

Full-length coding sequences of *CitERF13* and *CitVHA-c4* were amplified with primers (listed in Supplementary Table 3) and were constructed into pGreen II 0029 62-SK vector (SK). The detail information of the SK vector was described in Hellens *et al*.^27^. The constructs were electroporated into *Agrobacterium* GV3101. *Agrobacterium* cultures, carrying empty vector (SK), *CitERF13, CitVHA-c4* and *CitERF13* + *CitVHA-c4*, were infiltrated into different sites of same leaf, as indicated in Figure S1. Five days after infiltration, the infiltrated leaves were sampled and used for citric acid analysis.

Furthermore, transient over-expression was also conducted in *Arabidopsis* leaves, to verify the function of *AtERF017*, a homolog of *CitERF13*. Seeds of *AtVHA-c4* mutant (CS838548) were obtained from The *Arabidopsis* Information Resource (TAIR). Full-length of *AtERF017* was obtained with primers listed in Table S3 and was also constructed in the SK vector. Using the same protocol of tobacco system, *Agrobacterium*, carrying *AtERF017* and empty vector (SK), were transiently over-expressed in leaves of wild type (Col) and the two mutant lines. Infiltrated leaves were sampled and used for citric acid analysis.

The citric acid content of infiltrated leaves of *N. tabacum* and *Arabidopsis* were measured according Lin *et al*.^52^. Leaves (0.05 g) were ground in liquid nitrogen and extracted with 1.4 ml of methanol, at 70 °C for 15 min, and then centrifuged at 10,000 g. The upper phase was removed and stored at −80 °C until analysis. Aliquots of 100 μl upper phase were dried in vacuum. The residue was dissolved in 40 μl 20 mg ml^−1^ pyridine methoxyamine hydrochloride, and incubated at 37 °C for 1.5 h. The sample was then treated with 60 μl Bis (trimethylsilyl) trifluoroacetamide (1% trimethylchlorosilane) at 37 °C for 30 min. Ribitol (20 μl, 0.2 mg ml^−1^) was added into each sample as an internal standard. A 1 μl aliquot of each sample was absorbed with a split ratio of 1:1 and injected into GC-MS fitted with a fused-silica capillary column (30 m × 0.25 mm i.d., 0.25 μm DB-5 MS stationary phase). The injector temperature was 250 °C and the helium carrier gas had a flow rate of 1.0 ml min^−1^. The column temperature was held at 100 °C for 1 min, increased to 184 °C with a rate of 3 °C min^−1^, then increased to 230 °C with rate of 15 °C min^−1^, held for 1 min. The MS operating parameters were as follows: ionization voltage was 70 eV, ion source temperature was 230 °C and the interface temperature was 280 °C.

### Statistical Analysis

Least significant difference (LSD) was calculated by DPS 7.05 (Zhejiang University, Hangzhou, China). The statistical significance of differences was calculated using Student’s *t*-test.

## Additional Information

**How to cite this article**: Li, S.-j. *et al*. The Citrus transcription factor, CitERF13, regulates citric acid accumulation via a protein-protein interaction with the vacuolar proton pump, CitVHA-c4. *Sci. Rep*. **6**, 20151; doi: 10.1038/srep20151 (2016).

## Supplementary Material

Supplementary Information
