# Supplementary material for: The Citrus transcription factor, CitERF13, regulates citric acid accumulation via a protein-protein interaction with the vacuolar proton pump, CitVHA-c4
Source: Sci Rep. 2016 Feb 3;6:20151. doi: 10.1038/srep20151 (PMC4738278; doi:10.1038/srep20151)
Supplement: Supplementary Information [file srep20151-s1.pdf]

The Citrus transcription factor, CitERF13, regulates citric acid accumulation via a protein-protein interaction with the vacuolar proton pump, CitVHA-c4

Shao-jia Li[1,2,3,#], Xue-ren Yin[1,2,3,#], Xiu-lan Xie[1], Andrew C. Allan[4,5], Hang Ge[1], Shu-ling Shen[1] & Kun-song Chen[1,2,3,*]

Fig. S1

The transient over-expression assay in tobacco leaves.

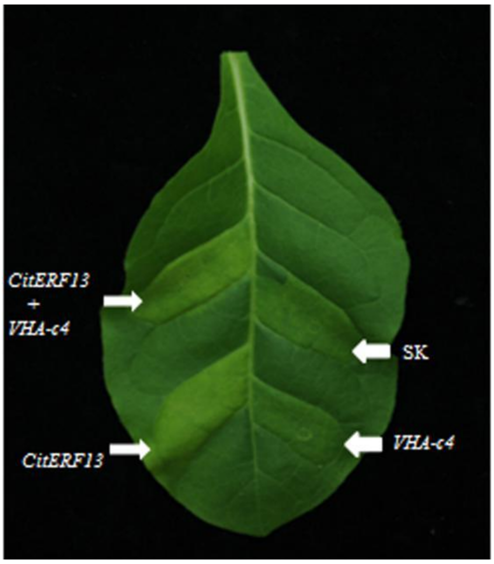

Fig.S2

Phylogenetic analysis of CitERF13 and CitVHA-c4. Genes of Arabidopsis ERF II Subfamily and VHA-c subunit were obtained from TAIR.

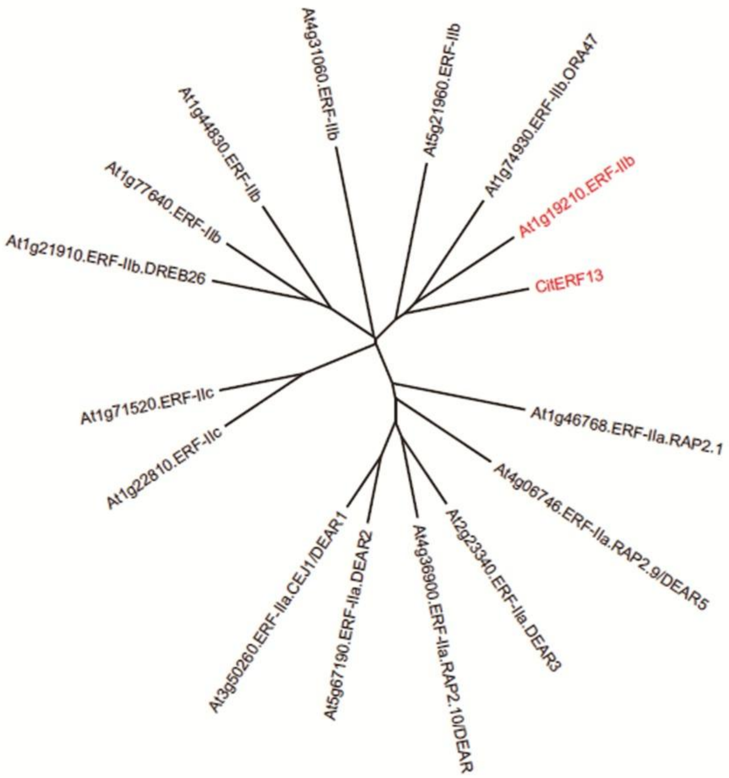

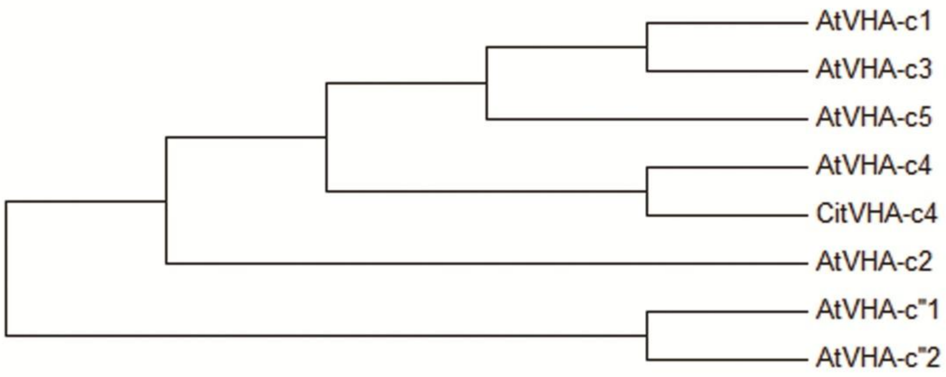

Fig. S3

Protein-protein interaction analyses using yeast two-hybrid assay.

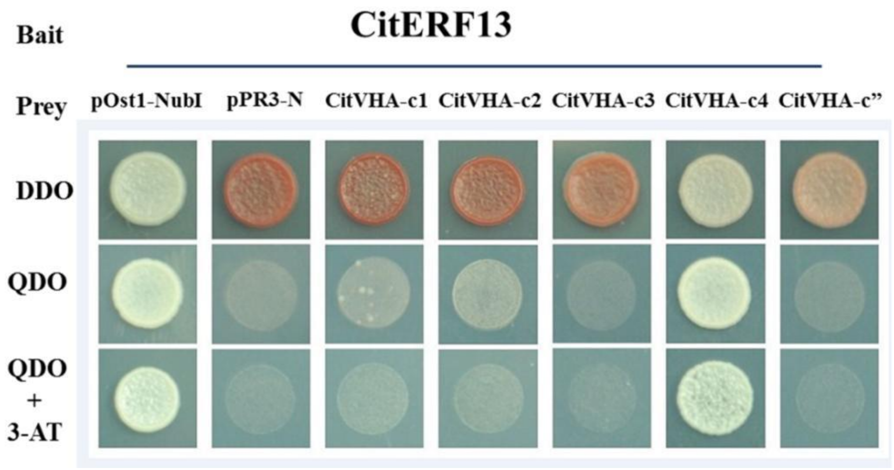

Fig. S4

Expression of the *CitERF13* and *CitVHA-c4* in flesh of ‘Gaocheng’ (GC) fruits and ‘Satsuma Mandarin’ (SM) during fruit development.

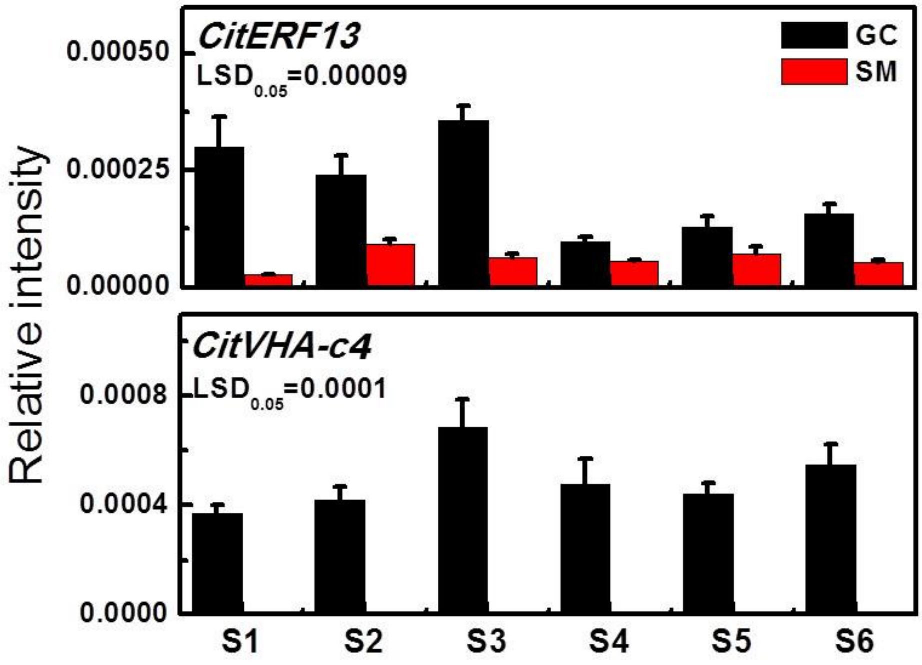

Table S1 Primers for real-time quantitative PCR analysis

Gene	Forward primer (5'-3')	Reverse primer (5'-3')
CitVHA-A1	AATGTTGGTGGGTCCATGTT	GAATTGAGGGAACGACAGGA
CitVHA-A2	CTCGCGACTGACAATCAAAT	CGGTACCAAACCAAAGATCG
CitVHA-B	TGTACCTTGCTGCCTCATTG	GGGCCTCAAGTGGACTACAA
CitVHA-C	GTTTTAGCTCCCTCCGTGAA	ATCATTCCGCCATCATCTTC
CitVHA-D	TGGATGAGCTTGAAAGAGAGG	TCCTTTTCCCCTGCAGATAA
CitVHA-E1	TTGAATTGTTTGTCACCCTCTG	CCACACCCCAAAAGATTTGA
CitVHA-F1	TCCTTTCCCTCGACTGCATA	TCCAAAGCCACCGTGTATTA
CitVHA-F2	CCCGCAATGTTCCATTTATC	CGAAGGAAGTTGAGCAAAGTG
CitVHA-G	CCTGGTTCGTTGTGAATCCT	TGCCCTAGGCAGATAATTTTG
CitVHA-H	ACAGTTTATCCAGTACCATCCAG	CGTACTTTGCGCCTAGGAAA
CitVHA-a1	GCTTTGGGATCTGTGCTCAT	ACTGTGCTCAGCAAACGATG
CitVHA-c1	TCTTGTAACGTTTATCCAGTCTGA	AAGGAAAACCAAGCCTGTGA
CitVHA-c2	ACTCTGGGGGAGCGAGATT	CTGCAAAAGTGCATGGACAG
CitVHA-c3	TTGGTGGTTATTTGGCCTGT	TCGAATCAATCTGGTTGAACA
CitVHA-c4	GGCGTGATTCAGAACTGAGG	CGTCGAGAACTCGAGCATTA
CitVHA-c”	GCTATATATGGGGTCATTGTTGC	TGCACAGCTGCTTCCAATTA
CitVHA-d	GGAGAGAGCCAAATGCTTGA	CATTCGGATATCCACATCAGG
CitVHA-e	GTTCTTGTGCAGAAGCTTATAGTATC	TCAAAGTTCATGCCCTCGAT
CitERF13	AGGAATGGACCATATGAGTGATG	TAGAAATTCCAGAGAAACGACGA
CitActin	CATCCCTCAGCACCTTCC	CCAACCTTAGCACTTCTCC
AtVHA-c4	TTCATGTGATGTGTAGCTTCTCC	CCGAGTCATATAATTTCACCCAAG
AtActin	AATGGAACTGGAATGGTCAAGGC	TGCCAGATCTTCTCCATGTCATCCCA

Table S2 Primers for yeast two hybrid and BiFC

Gene	Primers
CitVHA-c1	FP: AGAACGCGGCCATTACGGCCATGTCATCGACTTTCTCCGGCGACG
pDHB1	RP: CCGACATGGCCGAGGCGGCAATTCTGCCCGAGATTGGCC
CitVHA-c1	FP: GCAGAGTGGCCATTACGGCCATGTCATCGACTTTCTCCGGCGACG
pPR3-N	RP: CTCGAGAGGCCGAGGCGGCCGTTCTGCCCGAGATTGGCC
CitVHA-c2	FP: AGAACGCGGCCATTACGGCCATGCCTTCAACATTCAGCGGCGAT
pDHB1	RP: CCGACATGGCCGAGGCGGCAAATCTGCTCTGGACTGACCAGC
CitVHA-c2	FP: GCAGAGTGGCCATTACGGCCATGCCTTCAACATTCAGCGGCGAT
pPR3-N	RP: CTCGAGAGGCCGAGGCGGCCGATCTGCTCTGGACTGACCAGC
CitVHA-c3	FP: AGAACGCGGCCATTACGGCCATGTCTTCAACATTATTCAGTGGCG
pDHB1	RP: CCGACATGGCCGAGGCGGCAAGTCTGCTCTTGACTGACCAGC
CitVHA-c3	FP: AGAACGCGGCCATTACGGCCATGTCTTCAACATTATTCAGTGGCG
pPR3-N	RP: CTCGAGAGGCCGAGGCGGCCGGTCTGCTCTTGACTGACCAGC
CitVHA-c4	FP: AGAACGCGGCCATTACGGCCATGTCATCCGCGTTCATTGGC
pDHB1	RP: CCGACATGGCCGAGGCGGCAAATCTGCTCTGGATTGGCC
CitVHA-c4	FP: AGAACGCGGCCATTACGGCCATGTCATCCGCGTTCATTGGC
pPR3-N	RP: CTCGAGAGGCCGAGGCGGCCGATCTGCTCTGGATTGGCC
CitVHA-c”	FP: AGAACGCGGCCATTACGGCCATGTCGGGCTCCGTAATGTTG
pDHB1	RP: CCGACATGGCCGAGGCGGCAAGTCTGCTCTTGACTGACCAGC
CitVHA-c”	FP: AGAACGCGGCCATTACGGCCATGTCATCCGCGTTCATTGGC
pPR3-N	RP: CTCGAGAGGCCGAGGCGGCCACTGATGAGATTCTTGGAGG
CitERF13	FP: AGAACGCGGCCATTACGGCCATGGTGAAGACAACTGAAAAA
pDHB1	RP: CCGACATGGCCGAGGCGGCAAGAAATTCCAGAGAAACGA
CitERF13	FP: AGAACGCGGCCATTACGGCCATGGTGAAGACAACTGAAAAA
pPR3-N	RP: CTCGAGAGGCCGAGGCGGCCGGAAATTCCAGAGAAACGA
CitERF13	FP: CCCAAATTCGCGACCGGTATGGTGAAGACAACTGAAA
YN	RP: CTTGCTCACCATACCGGTGAAATTCCAGAGAAACGAC
CitERF13	FP: CCCAAATTCGCGACCGGTATGGTGAAGACAACTGAAA
YC	RP: GCTGCACGCTGCCACCGGTGAAATTCCAGAGAAACGAC
CitVHA-c4	FP: CCCAAATTCGCGACCGGTATGTCATCCGCGTTCATTG
YN	RP: CTTGCTCACCATACCGGTATCTGCTCTGGATTGGCC
CitVHA-c4	FP: CCCAAATTCGCGACCGGTATGTCATCCGCGTTCATTG
YC	RP: GCTGCACGCTGCCACCGGTATCTGCTCTGGATTGGCC

Table S3 Primers for transient over expression

Gene	Primers
CitVHA-c4	FP: AGGAGGCGGCCGCATGTCATCCGCGTTCATTG
	RP: AATCAACTAGTCTAATCTGCTCTGGATTGGCC
CitERF13	FP: CGGTGGCGGCCGCATGGTGAAGACAACTGAAAAAGAAC
	RP: GATCCACTAGTTTAGAAATTCCAGAGAAACGACGACG
AtERF017	FP: ATGGAAGGGTCGTCTTCTT
	RP: ATGGAATTCAAAAATTCC
